# Phosphate stimulates myotube atrophy through autophagy activation: evidence of hyperphosphatemia contributing to skeletal muscle wasting in chronic kidney disease

**DOI:** 10.1186/s12882-018-0836-2

**Published:** 2018-02-27

**Authors:** Yue-yue Zhang, Man Yang, Jin-fang Bao, Li-jie Gu, Hong-lei Yu, Wei-jie Yuan

**Affiliations:** Department of Nephrology, Shanghai General Hospital, Shanghai Jiaotong University School of Medcine, Haining Road 100rd, Hongkou Distrinct, Shanghai, 200080 China

**Keywords:** Hyperphosphatemia, Autophagy, Muscle wasting, Cell atrophy, Chronic kidney disease

## Abstract

**Background:**

Accelerated muscle atrophy is associated with a three-fold increase in mortality in chronic kidney disease (CKD) patients. It is suggested that hyperphosphatemia might contribute to muscle wasting, but the underlying mechanisms remain unclear. Although evidence indicates that autophagy is involved in the maintenance of muscle homeostasis, it is not known if high phosphate levels can result in activation of autophagy, leading to muscle protein loss.

**Methods:**

Immortalized rat L6 myotubes were exposed to a high concentration of phosphate, with or without autophagy inhibition. Myotube atrophy was examined by phase contrast microscopy. Autophagic activity was assessed by measuring the expression of microtubule-associated protein 1 light chain 3 (LC3) and p62 using quantitative real-time polymerase chain reaction and western blot.

**Results:**

Phosphate induced cell atrophy in L6 myotubes in a dose- and time-dependent manner, and these responses were not associated with calcification or osteogenesis. Phosphate also dose- and time-dependently increased the LC3-II/LC3-I ratio. Inhibition of autophagy with wortmannin or knockdown of *Atg5* significantly suppressed myotube atrophy caused by high phosphate concentration.

**Conclusions:**

High phosphate concentration induces muscle cell atrophy through the activation of autophagy. Targeting autophagy could be a therapeutic strategy for preventing muscle wasting caused by hyperphosphatemia.

## Background

Skeletal muscle wasting is thought to be one of the most effective markers of protein-energy wasting (PEW) in chronic kidney disease (CKD). Increased muscle atrophy is associated with a three-fold increase in 4–6-year mortality [1]. PEW referes to concurrent losses in protein and energy stores [2]. Multiple studies have now shown that PEW is closely related to major adverse clinical outcomes and leading to increased hospitalization rates and mortality in CKD patients, especially in maintenance hemodialysis treatment of end-stage renal disease [3].

Hyperphosphatemia, that is, serum inorganic phosphate (Pi) levels typically exceeding 2 mmol/L, is due to decreased Pi clearance in CKD relative to Pi overload. Hyperphosphatemia is very common in patients with CKD. Studies have investigated the influence of Pi overload on renal disease, cardiovascular disease, bone resorption, brain development, lung development, liver development, aging, and fertility [4]. *Klotho*-knockout mice develop severe hyperphosphatemia and have similar human aging phenotype, including muscle wasting, atherosclerosis, ectopic calcifications, and a very short life span. Moreover, the levels of Pi are normal in *NaPi2a*^−/−^ and *klotho*^−/−^ double-knockout mice, which indicated that the increase of bodyweight and lifespan compared to *klotho*-knockout mice [5]. This provides in vivo evidence for Pi toxicity accelerating the muscle wasting process and suggests a novel role for phosphate in muscle wasting. However, it is still unclear whether Pi overload can directly influence skeletal muscle cell metabolism.

There are also mechanistic studies on Pi toxicity, but these have mainly focused on its effects on vascular smooth muscle cell calcification, osteoblastic differentiation, and bone absorption [4]. Dai et al. [6] found that autophagy may be mediated by the endogenous protective mechanism to reduce matrix vesicle release and inhibit Pi-induced vascular calcification, but it is unclear whether autophagy is involved in the molecular mechanism of muscle atrophy induced by high Pi is unclear-.

Therefore, in this study we stimulated immortalized rat L6 cells with different Pi concentrations, to study the direct effect of Pi overload on skeletal muscle and its possible mechanisms of action. The aim of this work was to find a promising treatment for the prevention of PEW in CKD patients.

## Methods

### Cell culture and measurements of cellular size

Rat L6 myoblasts (Chinese Academy of Sciences,catalog numbers GNR4) were passaged as previously described [7, 8]. Briefly, cells were seeded into wells of 6-well plates at a density of 1 × 10^5^ cells per well in DMEM (Gibco, Gaithersburg, MD, USA) including 10% fetal bovine serum (Gibco) and antibiotics of 1% penicillin/streptomycin and incubated overnight. Cells were then incubated for 48 h in media with 2% horse serum instead of fetal bovine serum to induce differentiation. We then added appropriate amounts of sodium phosphate buffer (1 M Na_2_HPO_4_/NaH_2_PO_4_, pH 7.4) to achieve final Pi concentrations of 1.0, 2.0, and 3.0 mM. For the time-course experiment, L6 cells were incubated with medium containing 3 mM Pi for 3, 6, 12, and 24 h. The cells were cotreated with 100 mM wortmannin 100 (Selleck Chemicals, Houston, TX, USA) and transfected with either an *Atg5* knockdown plasmid **(**pLKO.1-EGFP-Atg5, Atg5−/−) or an *Atg5* overexpression plasmid (CD513B-1-Atg5, Atg5) in Optimem medium with Lipofectamine 2000, according to the manufacturer’s instructions, to inhibit and to induce autophagy, respectively. The cell size was measured from 6 random fields by Image-Pro Plus software (Media Cybernetics, Silver Springs, MD, USA).

### Von Kossa and alizarin red staining

Calcification was visualized by von Kossa (GENMED, USA, GMS80045.3) or Alizarin Red (GENMED, USA, GMS80046.3) staining, performed as described in the manufacturer’s instructions.

### Western blot analysis

The RIPA lysis buffer (Jierdun, China, BYL40825) used for L6 cells containing a protease inhibitor cocktail (Sigma-Aldrich, St Louis, MI, USA) and a phosphatase inhibitor mixture (PhosSTOP; Roche Applied Science, Indianapolis, IN, USA), the cells were treated at 4 °C for 30 min. After centrifuged at 12,000 g for 10 min at 4 °C, the supernatants of the lysate were transferred into separate tubes. SDS-PAGE were used to separate the protein which were then transferred onto nitrocellulose membranes, each volumes were 30 μg. The protein were then blocked by incubation overnight at 4 °C in 5% skim milk with primary antibodies. The antibodies used were as follows: microtubule-associated protein 1 light chain 3 (LC3; CST, Danvers, MA, USA; 2775 s); P62 (Abcam, Cambridge, MA, USA; Ab56416); GAPDH (CST, 5174); and H3 (CST, 4499 s). Membranes were washed and incubated using a secondary anti-rabbit IgG antibody (A0208, Beyotime Institute of Biotechnology, Shanghai, China), anti-goat IgG antibody (A0181, Beyotime Institute of Biotechnology), anti-mouse IgG antibody (A0216, Beyotime Institute of Biotechnology), or antibody conjugated with horseradish peroxidase (Beyotime Institute of Biotechnology). Bands were visualized using an ECL Western Blotting Substrate Kit (WBKLS0100; Millipore, Billerica, MA, USA).

### Quantitative real-time polymerase chain reaction (PCR)

Real-time PCR analysis to determine levels of specific mRNA transcripts in cultured cells were performed as described in references [8, 9]. Briefly, the Trizol was used to extract RNA. and cDNA libraries was prepared using 1 μg total RNA, which were then diluted 25-fold for real-time PCR using Taqman 2× PCR reagent and Taqman real-time PCR probe sets (Applied Biosystems, Foster City, CA, USA). RCAN1.4 probes were ordered through the Assays-by-Design service of Applied Biosystems. mRNA levels in each sample were tested three times and were calculated subsequent for the mean Ct. Data were normalized relative to the expression of 18S rRNA. Levels of mRNA were calculated according to appropriate controls under specific experiments and expressed as fold induction using the 2^−ΔΔCt^ method [10]. The primers used for real-time PCR are shown in Table 1.

**Table 1:** PCR primer sequences

Gene name	Forward primer (5′- 3′)	Reverse primer (5′- 3′)
*LC3*	CACTGCCGCCCTAAAGGTTAC	GACCGCTCCGTCAGAATGTTG
*p62*	TCCCTGTCAAGCAGTATC	GATCTTTGCACCAGTCTC
*BMP-2*	CTGTCCCTACTGATGAGTTTC	CTAACCTGGTGTCCAATAGTC
*OPN*	TATCCCGATGCCACAGATG	GTGTTTCCACGCTTGGTTC
*Osx*	CAAGGCAGTTGGCAATAG	AATGGGCTTCTTCCTCAG
*Pit-1*	AAACGGAGGACAACTATCAG	TACCACAGGCAAGTCTTATC
*ALP*	CCTTCCGGTATTGACTGTG	GAAACCTGACCCTGAAGTG
*Cbfa-1*	ACTTCGTCAGCGTCCTATC	CATCAGCGTCAACACCATC
*MGP*	AAAGCCCAGGAAAGAGTC	TGAAGTAGCGGTTGTAGG

*Abbreviations*: *LC3* Microtubule-associated protein 1 light chain 3, *ALP* Alkaline phosphatase, *BMP-2* Bone morphogenetic factor-2, *Cbfa1* Core-binding-factor α1, *MGP* Matrix GLA protein, *OPN* Osteopontin, *Osx* osterix

## Results

### Phosphate overload induces L6 cell atrophy

**Fig. 1:**
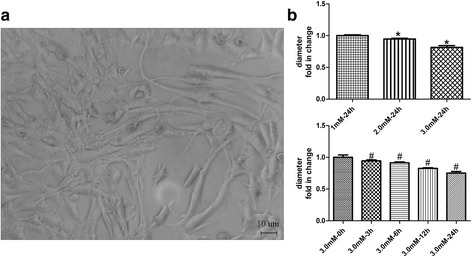
Phosphate overload induces L6 cells atrophy in a dose- and time-dependent manner. **a** Phase contrast microscopy showing cell morphology (Not every image is not shown here). **b** Statistics of cell diameter in each group. Data are shown as fold change compared with the control group (1 mM at 24 h and 3 mM at 0 h). *compared with 1 mM at 24 h, *p* < 0.05, # *p* < 0.05 versus 3 mM at 0 h

We examined the dose- and time-dependent effects of Pi on the diameter of L6 cells with phase contrast microscopy. L6 cells exposed to Pi at 2 mM and 3 mM resulted in a 5.4% and 13.0% decrease in cell diameter, respectively (*p*<0.05), compared with cells treated with 1 mM Pi for 24 h (control). The stimulatory effect of high Pi (3 mM) on cell diameter was observed at 3 h, 6 h, 12 h, and 24 h after treatment, with cell diameter decreasing 5.6%, 8.6%, 17.5%, 24.8% respectively (*p*<0.05, Fig. 1).

### Phosphate overload did not induce calcification or osteogenic differentiation

**Fig. 2:**
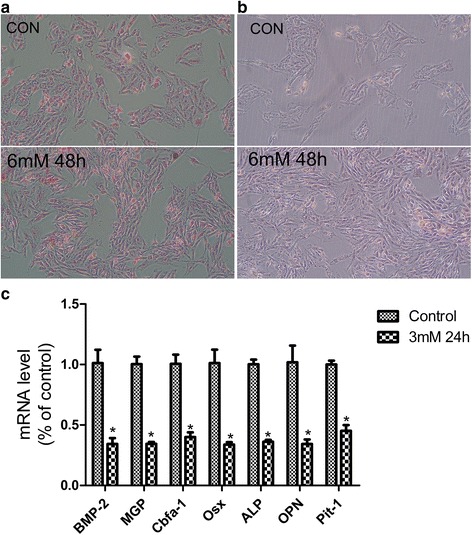
Phosphate overload did not induce calcification or osteogenic differentiation. **a** Von Kossa staining of L6 cells treated for 24, 48, or 72 h with control or high-Pi medium. Magnification = 100×. **b** Alizarin Red staining of L6 cells treated for 24, 48, or 72 h with control or high-Pi medium. Magnification = 100×. **c** Effect of high-Pi medium on the expression of bone morphogenetic protein-2 (BMP-2), osteopontin (OPN), osterix (Osx), Pit-1, core-binding-factor α1 (Cbfa1), ALP and matrix GLA protein (MGP) after 24 h. Expression levels were normalized to GAPDH and expressed relative to control medium. *P < 0.05 versus 3 mM at 0 h

We treated L6 cells with either control or high-Pi medium (3 mmol/L or 6 mmol/L Pi to model calcification), and detected calcification with the method of von Kossa stains (Fig. 2a) and Alizarin Red stains (Fig. 2b), but did not find significant calcification in high-Pi group. We also quantified RNA expression of the osteogenic differentiation factor, bone morphogenetic factor-2 (BMP-2); its inhibitor, matrix gamma carboxyglutamic acid (GLA) protein; the osteogenic transcription factors, Cbfa1 and osterix; the mineralization regulators, alkaline phosphatase (ALP; tissue-non-specific isoform) and osteopontin (OPN); Pit-1, the sodium-dependent Pi transporter by real-time PCR, as previous described in reference [11], to see whether high Pi induce osteogenic differentiation in L6 cells, and did not find high Pi induce significant increase of any of these genes at 24 h (Fig. 2c).

### Phosphate overload is associated with increased autophagy

**Fig. 3:**
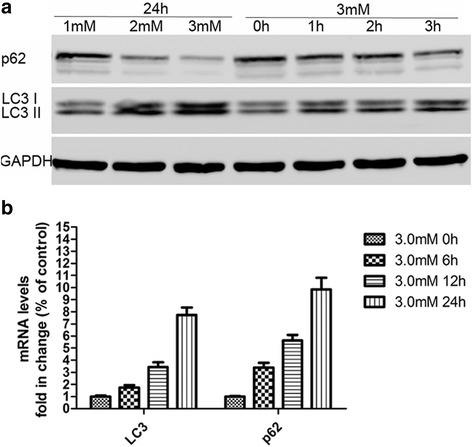
Phosphate overload is associated with increased autophagy. **a** Western blots show the ratio of LC3II/I increasing in a dose- and time-dependent manner, while p62 protein decreases, indicating autophagy upregulation. **b** mRNA levels of *p62* and *LC3* increase with increased phosphate treatment time. Data are shown as fold change compared with the control group (3 mM at 0 h). *p < 0.05 versus 3 mM at 0 h

Exposure of L6 cells to Pi at 2 mM and 3 mM resulted in a 1.9-fold and 2.6-fold increase in the LC3-II/LC3-I ratio, respectively, compared with cells treated with 1 mM Pi for 24 h. The stimulatory effect of high Pi (3 mM) on the LC3-II/LC3-I ratio was observed as early as 3 h after treatment. In contrast, the levels of p62 decreased (Fig. 3a). We further examined the time-dependent effects of Pi on *LC3* and *p62* mRNA transcription, and found *LC3* and *p62* increased with prolonged exposure to 3 mM Pi (Fig. 3b).

### Blockage of autophagy attenuated high pi-induced cell atrophy in L6 cells

**Fig. 4:**
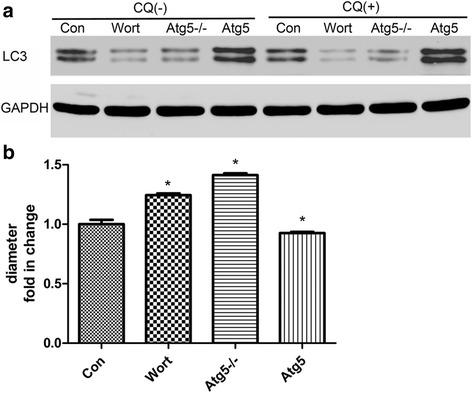
Blockage of autophagy attenuated high Pi-induced cell atrophy in L6 cells. **a** Western blot shows the ratio of LC3II/I decreases with wortmannin or pLKO.1-EGFP**-**Atg5 (Atg5 −/−) treatment and increases with CD513B-1-Atg5 (Atg5) treatment. **b** Cell diameter with different treatments. Data are shown as fold change compared with the control group (3 mM, 24 h). *p < 0.05 versus control

We added the pharmacologic inhibitor of autophagy, wortmannin, and the Atg knockdown plasmid, pLKO.1-EGFP-Atg5, to the culture medium in the presence of a high concentration of Pi. The Agt5 overexpression plasmid, CD513B-1-Atg5 was used as a positive control. As shown in Fig. 4, wortmannin and pLKO.1-EGFP-Atg5 significantly attenuated the Pi-induced LC3-II/LC3-I ratio. The diameter of L6 cells treated with 3 mM Pi plus wortmannin or pLKO.1-EGFP-Atg5 was larger than cells treated with 3 mM Pi only.

## Discussion

In this study, we found that the incubation of L6 cells with high Pi-containing medium induced cell atrophy in a time- and dose-dependent way, indicating that Pi overload could affect skeletal muscle cells directly. Most previous studies have examined the effect of Pi on calcification or osteogenic differentiation. Prior studies in vascular smooth muscle cells (VSMC) have shown an important role for BMP-2, OPN, and other osteogenic markers in phosphate-induced VSMC calcification [11]. We tested the effect of high-Pi medium on calcification and osteogenic differentiation of L6 cells, but found no significant effect.

Autophagy, the lysosomal-dependent cellular turnover of organelles and proteins, has been shown to play a crucial role in muscle atrophy. The autophagy-lysosome system is a proteolytic pathway that mainly degrades long-lived proteins and organelles, such as mitochondria and the sarcoplasmic reticulum. It operates alongside the ubiquitin-proteasome system, which has been shown to play an important role in muscle atrophy and its activation has been well documented in CKD-associated muscle atrophy [12, 13]. There are roughly three classes of autophagy: macroautophagy, microautophagy, and chaperone-mediated autophagy. In macroautophagy (hereafter referred to as autophagy), membrane vesicles form autophagosomes by sequestering a small portion of the cytoplasm. These autophagosomes then fuse with lysosomes to degrade the materials within them [14]. In recent years, the activation of skeletal muscle autophagy has been demonstrated under various conditions and disease states ranging from fasting [15, 16], oxidative stress [17], denervation [18, 19], and drug effects [20, 21] to systemic diseases, such as sepsis [22], merosin-deficient congenital muscular dystrophy (MDC1A) [23], and cancer [16]. In a previous study, we reported the upregulation of mRNA and protein expression of Bnip3 and LC3B in the rectus abdominis of CKD patients [24]. Thus, in this study, we investigated whether Pi overload induces cell atrophy through autophagy upregulation. During autophagosome formation, the cytosolic LC3-I protein is converted to LC3-II through lipidation. Western blot analysis showed that high Pi induces a dose- and time-dependent increase in the LC3-II/LC3-I ratio, which has been widely used as an indicator of autophagic activity. The levels of p62, a protein known to be incorporated into autophagosomes and efficiently degraded, decreased with high Pi. While PCR analysis of *LC3* and *p62* mRNA also suggested a transcription mechanism of Pi effect. We further used a pharmacological agent and genetic method to study the effect of autophagy on cell atrophy. The inhibition of autophagy by wortmannin or Atg5 knockdown significantly inhibited high Pi-induced atrophy. These findings indicate a stimulatory effect of high Pi on autophagy in skeletal muscle and suggest that autophagy may participate in the development of muscle atrophy induced by high Pi in CKD.

A low protein diet has been recommended for patients with CKD to maintain or improve nutritional status [25]. Other studies have found that a very-low-protein diet [26] or a low protein diet supplemented with ketoacids [27, 28] can also improve calcium and phosphorus metabolism. Together with our previous report [29], we hypothesize that hyperphosphatemia is an independent risk factor of CKD skeletal muscle wasting and proper low Pi diet may be a promising approach to maintain or improve skeletal muscle wasting in CKD patients. However, further studies are still needed to confirm this hypothesis.

Yamada et al. [30] used dietary Pi overload to induce hyperphosphatemia in rats with CKD. serum albumin level, body weight, and muscle mass in these rats were decreased by Pi-overload-related chronic inflammationassociated with. However, there are still conflicting results on the effect of hyperphosphatemia in CKD skeletal muscle wasting in vivo. This study is only a preliminary study on Pi toxicity in skeletal muscle cells, being limited to in vitro experiments. Further studies are still needed to investigate the in vivo effects of adjusting Pi levels.

## Conclusion

Our results indicate that a high concentration of phosphate induces protein loss in L6 myotubes. This response is associated with an induction of autophagic activity. Notably, inhibition of autophagy significantly suppresses the detrimental effect of phosphate in myotubes, suggesting that activation of autophagy mediates high phosphate-induced myotube atrophy. Thus, these results suggest that an increase in autophagic activity plays a critical role in the pathogenesis of muscle wasting occurring in CKD or patients with hyperphosphatemia.

## Abbreviations

ALP: Alkaline phosphatase
BMP-2: Bone morphogenetic factor-2
Cbfa1: Core-binding-factor α1
CKD: Chronic kidney disease
LC3: Microtubule-associated protein 1 light chain 3
MDC1A: Merosin-deficient congenital muscular dystrophy
MGP: Matrix GLA protein
OPN: Osteopontin
Osx: Osterix
PEW: Protein-energy wasting
Pi: Inorganic phosphate
VSMC: Vascular smooth muscle cell
